# Nutri-Epigenetic Regulation of Vitamin D—Impact on Metabolism and Biological Functions: Narrative Review

**DOI:** 10.3390/metabo15070436

**Published:** 2025-06-30

**Authors:** Magdalena Kowalówka, Ilona Górna, Marta Karaźniewicz-Łada, Dominika Kusyk, Juliusz Przysławski, Sławomira Drzymała-Czyż

**Affiliations:** 1 Department of Bromatology, Faculty of Pharmacy, Poznan University of Medical Sciences, 60-806 Poznan, Poland; igorna@ump.edu.pl (I.G.); 87786@student.ump.edu.pl (D.K.); jprzysla@ump.edu.pl (J.P.); drzymala@ump.edu.pl (S.D.-C.); 2 Department of Physical Pharmacy and Pharmacokinetics, Faculty of Pharmacy, Poznan University of Medical Sciences, 60-806 Poznan, Poland; mkaraz@ump.edu.pl

**Keywords:** gene expression, epigenome, polymorphism, nutrigenomics, vitamin D response index

## Abstract

Vitamin D deficiency is widespread. It increases the risk of several diseases. Therefore, researchers have long studied the factors that influence vitamin D levels in the body. These include its metabolism, catabolism, transport and binding of vitamin D to the receptor VDR. Currently, an increasing number of studies are focusing on genetic factors. Variations in vitamin D levels, including vitamin D deficiency, are under substantial genetic control. There is a reciprocity between the vitamin D system and epigenetic mechanisms. Vitamin D metabolism, on the one hand, is regulated by epigenetic mechanisms and, on the other hand, is involved in regulating epigenetic events. To appraise recent advances in nutrigenomics with its application in public health, several databases, including PubMed, Scopus and Web of Science, were investigated in detail. Nutri-epigenetics deals with the interplay between dietary components and the possible resulting changes in the epigenome. There is, therefore, great potential for the development of nutri-epigenetics. The purpose of the narrative review is to highlight the genetic aspects of vitamin D, its receptor VDR and vitamin D-related gene polymorphisms with a particular focus on vitamin D gene regulation. Particular attention is paid to the vitamin D response index.

## 1. Introduction

Vitamin D is a key regulator of calcium–phosphorus homeostasis and bone health and is essential in numerous metabolic and immune processes. Its action, however, goes far beyond classic hormonal functions, including regulating gene expression through epigenetic mechanisms. Epigenetics refers to heritable changes in gene function that occur without changes in the DNA sequence.

Processes such as DNA methylation, histone modifications and the influence of microRNA can be modulated by environmental factors, including dietary components. In the context of vitamin D, the so-called nutri-epigenetics, which studies the interactions between nutrients (np, vitamin D) and the epigenome that influence the expression of genes involved in regulating metabolism and biological functions, is particularly interesting.

Moreover, growing evidence supports the vitamin D response index concept, which reflects interindividual variability in responsiveness to vitamin D based on genetic and epigenetic backgrounds. This variability may explain why some individuals respond strongly to supplementation while others do not achieve significant biological benefits despite adequate serum 25(OH)D levels. Understanding the epigenetic landscape of vitamin D metabolism has implications for personalised nutrition and targeted supplementation strategies. Despite numerous studies on vitamin D’s role in health, there remains limited integration of findings on its epigenetic influence and its implications for personalised supplementation strategies.

The narrative review aims to present the current knowledge on the role of vitamin D in epigenetic regulation and its impact on metabolic and physiological processes. Particular attention is paid to the molecular mechanisms underlying this regulation and the vitamin D response index.

## 2. Methods

### 2.1. Search Strategy

The study is guided by the Scale for the Assessment of Narrative Review Articles (SANRA) [1]. The international electronic databases including PubMed (Medline), Scopus and Web of Science were searched until 31 February 2025, by using appropriate keywords: “vitamin D” or “25(OH)D”, “regulation of vitamin D”, “epigenome”, “polymorphism”, “nutrigenomics” and “vitamin D response index”.

### 2.2. Inclusion and Exclusion Criteria

Eligibility is determined independently by reviewing whether the article fulfils the requirements based on the following criteria: (1) original study articles, (2) open access and in English, and (3) years of publication between 2000 and 2025. Afterwards, the full text of the articles was screened based on the inclusion and exclusion criteria.

Duplicate publications and studies without enough data and information were excluded. All potential articles were included and screened in two stages of eligibility. In the first assessment stage, screening was carried out by selecting titles and abstracts. The second stage was filtered based on the results and discussion under the study topic, and a comprehensive and independent analysis was performed.

Two expert reviewers independently assessed studies to evaluate their eligibility for inclusion.

## 3. Development

### 3.1. The Influence of Intracellular Factors and Environmental Stimuli on Epigenetic Modifications

**Figure 1:**
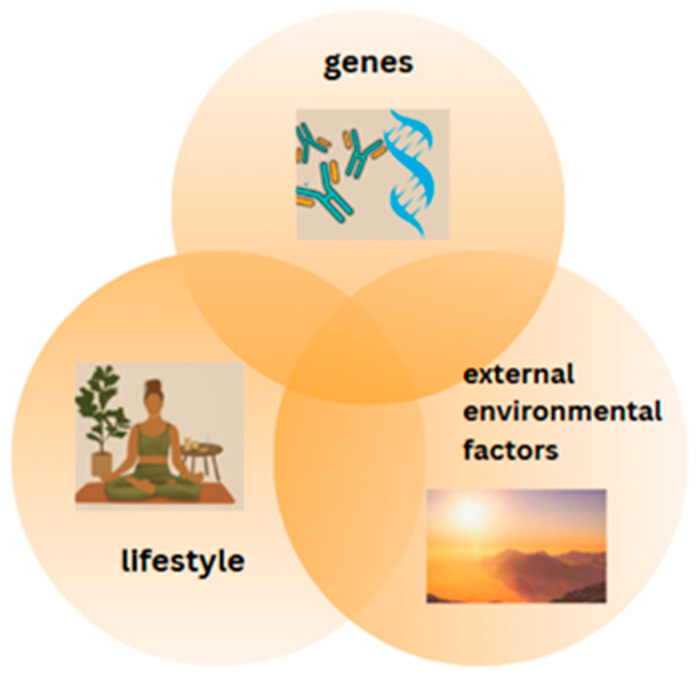
The relationship between genes, lifestyle choices and external environmental factors in shaping health. These three components interact with one another and collectively influence overall health outcomes.

Many diseases are caused by interactions between genes and environmental factors (such as diet). This relationship is the subject of nutrigenomics, which attempts to answer the following question: How does the composition of food, through changes in the expression and/or structure of the genome, influence the state between health and disease and, at the same time, to what extent does the individually diverse genome enable food components to affect health? Human health, therefore, is determined by several factors, which include environment, lifestyle and genetic factors (Figure 1). Lifestyle includes diet, physical activity, stress levels and rest.

Extragenic inheritance of traits refers to epigenetics, which deals with changes in chromatin organisation and gene expression that are not caused by changes in the DNA sequence but impact the inheritance of traits. This includes DNA methylation and covalent modifications of histones through methylation, acetylation, phosphorylation or ubiquitination. The interaction of different epigenetic mechanisms occurs through the presence of enzymes in the nucleus, i.e., DNA methyltransferase (DNMT) and translocation protein (TET), and several enzymes responsible for regulating histone modifications: histone acetyltransferases (HAT), histone deacetylases (HDAC), histone methyltransferases (HMT) and histone demethylases (HDM) [2].

**Figure 2:**
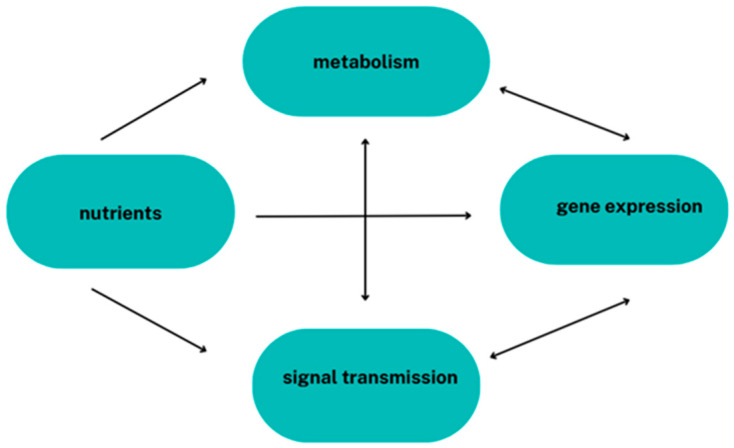
The influence of nutrients on gene expression.

The environment and human lifestyle significantly influence epigenetic modifications. The influence of diet on gene expression lies at the heart of nutrigenomics. The interplay between dietary intake components and possible resulting changes in the epigenome is defined by nutri-epigenetics. These sciences analyse the effects of bioactive dietary components on gene expression, i.e., investigating the relationship between diet and genetic predisposition to diet-related diseases and uncovering the mechanisms that determine how food components and nutrition affect health. There is currently great hope for the development of these sciences. Food components can directly and indirectly influence gene expression (Figure 2).

At the cellular level, some of the food components act as ligands for transcription factor receptors or enter metabolic pathways by altering the levels of intermediates and terminals, as well as modulating signal transduction pathways [3].

**Figure 3:**
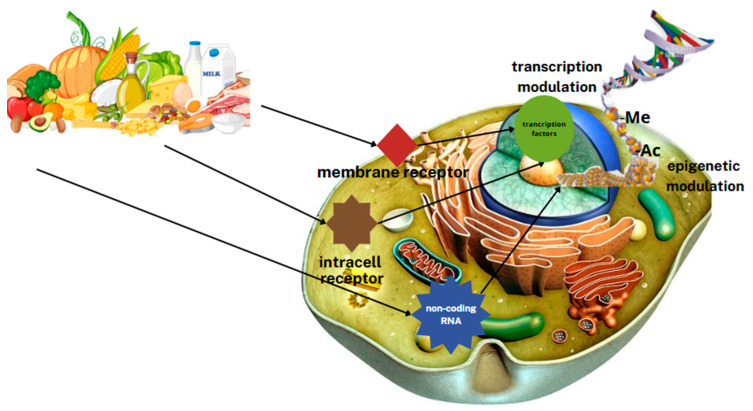
Effect of nutrients on gene expression. (-Ac—acetyl group in acetylated histones, -Me—methyl group in methylated DNA).

Bioactive dietary components can affect gene expression levels directly as nuclear receptor regulatory factors or indirectly through modifications of chromatin packing, such as DNA methylation (Figure 3) [4].

Studies of gene–nutrient interactions are based on molecular testing methods to identify single-nucleotide polymorphisms (SNPs), the phenotypes of which may lead to specific nutrient deficiencies or other physiological responses [5,6].

The effect of nutrients on the balance of genome methylation is thought to be one of the mechanisms preventing promoter hyper- or global hypomethylation. The impact on DNA methylation of components such as folate, vitamins B12 and B6, and polyphenols, e.g., epigallocatechin gallate and genistein, methionine, selenium and zinc, is now known to depend on the level of their intake. Quantitative and metabolic abnormalities of the above-mentioned components in the body can lead to DNA hypo-/hypermethylation, which can result in inappropriate gene expression and genetic instability and, ultimately, the development of a number of diseases [5].

Additionally, the effect of vitamin D on DNA methylation is considered. Severe vitamin D deficiency is associated with changes in leukocyte DNA methylation, and individuals with vitamin D deficiency are more likely to show reduced synthesis and increased catabolism of active vitamin D [7,8].

Vitamin D is a steroidal chemical that plays an important role in calcium homeostasis in the body. In addition, this vitamin is known to have a broad spectrum of action in various extraskeletal pathways. An adequate level of 25-hydroxyvitamin D (25(OH)D) in the body contributes to maintaining normal body weight and prevents obesity [9,10]. It regulates the immune system, participates in the detoxification of bile acids and plays a significant role in intestinal homeostasis. Vitamin D is one of the key controllers of systemic inflammation, oxidative stress and mitochondrial respiratory function, and thus, the human ageing process [11]. The molecular and cellular actions of 1,25 dihydroxyvitamin D (1,25(OH)2D) slow down oxidative stress and cell and tissue damage. On the other hand, vitamin D deficiency impairs mitochondrial function and increases oxidative stress and systemic inflammation [2]. The interaction of 1,25(OH)2D with its intracellular receptors modulates vitamin D-dependent gene transcription and activation of vitamin D-responsive elements, which triggers multiple second messenger systems [11]. The main causes of vitamin D deficiency include insufficient exposure to sunlight, a diet low in vitamin D-rich foods and lack of supplementation. In addition, certain medical conditions—such as intestinal, liver or kidney diseases—may impair the absorption and metabolism of vitamin D, further contributing to deficiency in specific patient groups. Hypovitaminosis D resulting from malabsorption or insufficient metabolism appears to generate the development of many diseases related to the skeleton, i.e., rickets and osteomalacia, as well as conditions affecting the central nervous system and other organs, including neurodegenerative and psychiatric diseases, autoimmune, cardiovascular, metabolic disorders and some cancers [12,13,14,15]. Understanding vitamin D-related advances in metabolomics, transcriptomics and epigenetics may provide better clinical outcomes in humans.

### 3.2. Metabolism of Vitamin D

**Figure 4:**
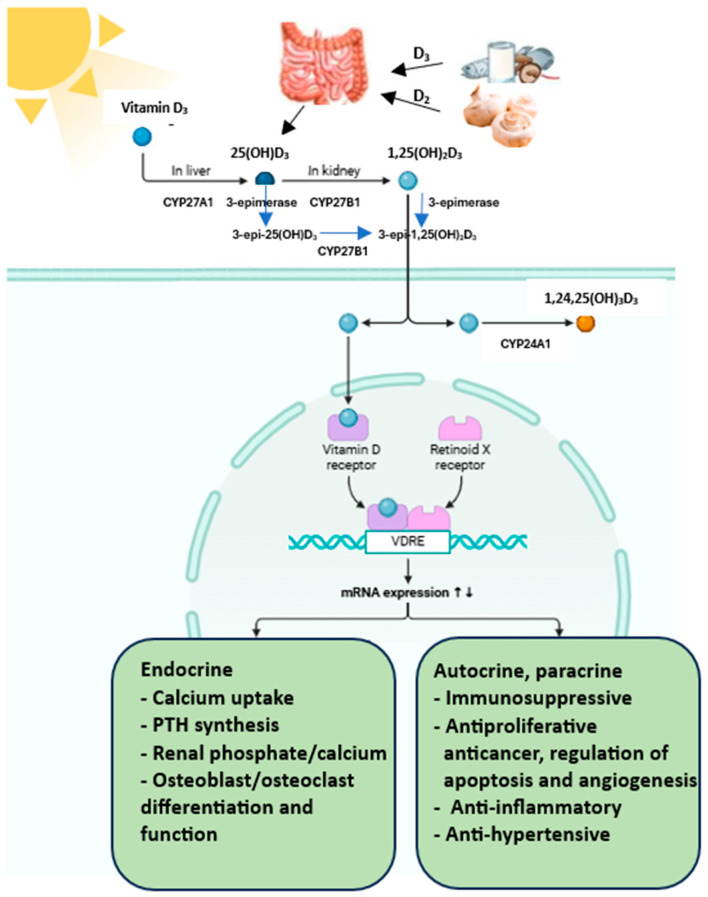
Vitamin D metabolism. CYP27A1 (25–hydroxylase), CYP27B1 (1α-hydroxylase), CYP24A1 (24-hydroxylase). Created in BioRender.com (accessed March 2025).

Many factors modulate vitamin D metabolism and function. Although serum vitamin D concentrations are highly dependent on diet and supplement intake and environmental factors, i.e., latitude and skin sun exposure, season, age and sex, they are also determined by genetic background [16]. Ergocalciferol (vitamin D2) and cholecalciferol (vitamin D3) are forms of vitamin D taken in foods and dietary supplements and produced in the skin (Figure 4). Exogenous sources of vitamin D are fatty fish, eggs and cheese (D3), mushrooms and yeast (D2), fortified foods and dietary supplements (D2 and D3).

However, the major source of vitamin D is its synthesis in unprotected skin from provitamin D3—7-dehydrocholesterol (7DHC) when exposed to UVB radiation, mainly in the range of 290–320 nm [17,18,19]. 7DHC is an intermediate compound in cholesterol synthesis. Vitamin D synthesis depends on the concentration of 7DHC, which in turn depends on the activity of 7-dehydrocholesterol reductase (DHCR7). This enzyme catalyses the reversible reduction of 7DHC to cholesterol [20].

7DHC undergoes non-catalytic thermal isomerisation to cholecalciferol. This is a biologically inactive compound [21]. Food-derived vitamin D3 is absorbed in the proximal part of the small intestine and transported in chylomicrons that enter the bloodstream via the lymph, while the form produced in the skin is transported in the blood with vitamin D-binding protein (VDBP) and/or albumin to the liver [22]. It has been shown that vitamin D is best absorbed when it is consumed with fat-rich food [23]. In hepatocytes, cholecalciferol or ergocalciferol is hydroxylated to 25(OH)D (calcidiol) by the microsomal hydroxylase CYP2R1 (a group of hydroxylases included in cytochrome P450) or D3 by the mitochondrial hydroxylase CYP27A1 [24]. Calcidiol is the primary metabolite of vitamin D, and its plasma concentration is considered the primary indicator of the body’s supply in clinical practice.

The second hydroxylation reaction takes place in the capillaries of the renal proximal tubules. With the participation of the mitochondrial enzyme CYP27B1 with a 1α-hydroxylase function (cytochrome P450 family), 25-hydroxy derivatives are converted to the active forms, dihydroxy derivatives of vitamin D, viz., 1,25(OH)2D3 and 1,25(OH)2D2 [24,25,26]. These forms bind to the vitamin D receptor and have the ability to exert biological effects. In contrast to hepatic 25-hydroxylases, renal 1α-hydroxylase is regulated by parathormone (parathyroid hormone, PTH), fibroblast growth factor 23 (FGF23) and 1,25(OH)2D3. PTH stimulates, while FGF23 and calcitriol inhibit CYP27B1.

Elevated calcium concentrations inhibit CYP27B1 mainly through PTH suppression, whereas higher phosphate levels inhibit CYP27B1 through FGF23 stimulation [27,28,29]. 1,25(OH)2D3 also directly inhibits CYP27B1 expression in the kidney through a mechanism involving the vitamin D receptor (VDR) and vitamin D inhibitor receptor (VDIR), which brings histone deacetylases (HDACs) and DNA methyltransferases to the Cyp27B1 promoter, ultimately inhibiting its transcription [24]. Hence, low calcium and phosphate intake, hypocalcaemia and a consequent increase in PTH result in active vitamin D production [28]. In contrast, increased levels of calcitriol inhibit PTH secretion and CYP27B1 activity.

The kidney is also a site of vitamin D catabolism; 25(OH)D3 and 25(OH)D2 can be hydroxylated at the 24th carbon atom by the cytochrome P450 enzyme, CYP24A1. The 24,25-dihydroxyvitamin D is formed, viz., 24,25(OH)2D3 and 24,25(OH)2D2. The CYP24A1 enzyme can also reduce the high amount of circulating 1,25(OH)2D3 and 1,25(OH)2D2 by their hydroxylation to 24-hydroxy products or 23-hydroxy derivatives. This process initiates the metabolic step of decomposing calcidiol and calcitriol into inactive products and are excreted in the bile or urine [30]. Oxidation of 1,25(OH)2D at the 24th carbon atom leads to the formation of 1,24,25(OH)3D, followed by calcitroic acid. Oxidation at the 23 carbon atom results in the formation of 1,23,25(OH)3D, followed by 1,25(OH)2D-23,26-lactone [25]. In addition to the classical metabolic pathway, vitamin D can undergo additional transformations under the action of the enzyme 3-epimerase (Figure 4). The activity of this enzyme has been detected in keratinocytes, parathyroid gland cells, osteoblasts, liver cells and colon cancer cells [31].

It causes a configuration change of the hydroxyl group at the C3 carbon atom from the alpha to the beta position: 25(OH)D2 is converted in the liver to 3-epi-25(OH)D2 and 25(OH)D3 to 3-epi-25(OH)D3 [31]. The same enzyme also catalyses the epimerisation of 1,25(OH)2D3 to 3-epi-1,25(OH)2D3 in the kidney. Vitamin D epimers show a lower affinity for DBP and VDR while maintaining their biological functions. The identical molecular weight and similar chemical properties of the epimeric and non-epimeric forms mean that high concentrations of 3-epi-25(OH)D3 may falsely overestimate measurements of 25(OH)D3 content in the body. On average, 3-epi25(OH)D3 accounts for 6.1% of the total 25(OH)D pool in the body in adults and 21.4% in infants [32]. Due to the low, often indeterminate levels of 25(OH)D2, 3-epi-25(OH)D2 does not significantly affect total body vitamin D levels [32]. 3-epi-1,25(OH)2D3 affects gene expression similarly to 1,25(OH)2D3 but exhibits a lesser biological effect due to its lower affinity for the VDR. It inhibits cell proliferation at a level of 30% and induces cell differentiation at 10% compared to the non-epimeric form. However, the ability of 3-epi-1,25(OH)2D3 to inhibit PTH secretion remains unchanged [31]. The resulting calcitriol binds to the carrier protein VDBP in serum and is transported to target tissues serum, and 15% is bound to albumin [30,33]. The lipophilic structure of this vitamin and its metabolites allow the molecules to pass through cell membranes. Thus, gene regulation by vitamin D is more direct and less complex than other hormones and signalling molecules [34]. Calcitriol mainly acts via the VDR receptor located in the cell nucleus. Since the VDR is the only 1,25(OH)2D3-binding protein with high affinity, the physiological effects of vitamin D are essentially identical to those of its receptor [34].

### 3.3. Genetic Determinants of Vitamin D Metabolism/Key Genes Associated with Vitamin D Metabolism

#### 3.3.1. VDR Receptor

Vitamin D action in cells requires the presence of cytosolic and nuclear VDR receptors. VDR regulation is complex and involves vitamin D, genetics and epigenetics. These three factors can interact to influence VDR function. The nuclear receptor is an activated ligand that acts as a transcription factor, encoding proteins that bind sequentially to genomic DNA and can modulate RNA polymerase II activity in gene expression [35].

The VDR belongs to a superfamily of steroid hormone receptors that includes receptors for retinoic acid, sex hormones and adrenal steroids, as well as thyroid hormones, and hence, 1,25(OH)2D3, like the steroid hormones, is active already at nanomole and even picomole concentrations [36].

The *VDR* gene is located on the long arm of chromosome 12 (12q12-q14) and consists of three main domains: a linker (hinge domain, HD), a DNA-binding domain (DBD) and a ligand-binding domain (LBD). Evidence exists for transcriptional modulation of the VDR through multiple signalling pathways in different cell types [37,38].

There are two pathways of calcitriol action through the VDR: genomic and non-genomic.

The genomic actions of vitamin D depend on the activation of the VDR by calcitriol and involve changes in the epigenome, which then lead to changes in the transcriptome and proteome. In the classical genomic response, 1,25(OH)2D3 binds to the VDR located in the cytoplasm. This association results in heterodimerisation with the retinoid X receptor (RXR) and binding of available genomic DNA. The preferred binding sites for the VDR-RXR complex are the RGKTSA sequence motifs (R = A or G, K = G or T, S = C or G) arranged as a direct repeat with three spacing nucleotides (DR3) [36,39]. Once translocated to the cell nucleus, the resulting heterodimer initiates transcription of vitamin D3-dependent genes in such a way that it binds to vitamin D response elements (VDREs). This, in turn, is made possible by the induction of an appropriate conformation of the DBD, causing it to become complementary to specific nucleotide sequences within the VDREs (Agonists of vitamin D—mechanisms of action selectivity). The formed complex of VDR, RXR and the VDRE sequence also interacts with nuclear coregulatory molecules that affect histone proteins, chromatin or type II RNA polymerase binding, directly affecting the gene transcription process [40]. VDR DNA binding is promoted by the pioneer factor PU.1 (purine-rich field). VDR receptors are present in more than 30 tissues and organs of the human body [41]. These include regions related to the body’s mineral metabolism and areas that do not have regulatory functions in this domain. The response to calcitriol at the genomic level is delayed and occurs after a time calculated in hours or days [41].

Chromatin immunoprecipitation sequencing (ChIP-seq), a next-generation method, can determine the genome-wide binding pattern (cistrom) of a transcription factor such as VDR. Based on ChIP-seq for VDR, it has been shown that DR3-type binding sites are the most enriched sequence motifs downstream of VDR peaks [36,42]. However, not all VDR-containing nuclear complexes contact genomic DNA via DR3 sites within a certain distance [42]. The low percentage of VDR complexes binding DR3 suggests a range of scenarios where the VDR acts independently of the RXR [36].

The second, non-genomic mechanism of action of vitamin D_3_ involves its interaction with membrane-associated vitamin D receptors (mVDR), located in specific regions of the plasma membrane such as caveolae or lipid rafts [38]. The calcitriol-binding site, in this case, is an alternative pocket within the VDR, displaced from its binding site in genomic action [42].

Non-genomic actions are manifested, in the main, as the activation of signalling molecules, such as phospholipase C and phospholipase A2 (PLA2), phosphatidylinositol-3 kinase (PI3K) and p21ras, and the rapid generation of second messengers (Ca^2+^, cyclic AMP, fatty acids and 3-phosphoinositides such as phosphatidylinositol 3,4,5 trisphosphate), accompanied by the activation of protein kinases, such as protein kinase A, src, mitogen-activated protein (MAP) kinases, protein kinase C (PKC) and Ca^2+^-calmodulin kinase II [43]. The non-genomic actions of vitamin D occur very rapidly (within seconds to minutes) and do not involve the VDR or changes in gene expression [44].

In some cell types, the non-genomic actions may also involve interaction with other membrane proteins, such as PDIA3 (also known as ERp57). The specific pathways activated vary depending on the target tissue and cellular context [44].

1,25(OH)2D controls genes related to energy metabolism, detoxification, immune function and maintenance of calcium homeostasis [45,46].

A serum vitamin D concentration of less than 50 nM (20 ng/mL) is considered insufficient [47]. Low vitamin D levels increase the risk of many diseases. The normal vitamin D level, according to the Endocrine Society, based on the concentration of 25(OH)D3, is assumed to be 75–100 nM (30–50 ng/mL) of 25(OH)D3 [48,49,50].

#### 3.3.2. *NADSYN1/DHCR7*

The *DHCR7* gene, located on chromosome 11q12–q13, encodes 7-dehydrocholesterol reductase, a key enzyme that converts 7-dehydrocholesterol (7-DHC) to cholesterol [51]. This reaction regulates the balance between cholesterol and vitamin D_3_ synthesis, as 7-DHC also serves as a precursor for vitamin D_3_ in the skin. The *NADSYN1* gene, located nearby, encodes nicotinamide adenine dinucleotide synthetase. DHCR7 may switch between cholesterol and vitamin D synthesis [51]. Cholesterol reciprocally affects DHCR7, increasing vitamin D production in skin cells [52]. Vitamin D can impede cholesterol synthesis by reducing DHCR7 protein expression dose-dependently [53]. The effect of the 7DHC enzyme on vitamin D3 biosynthesis has only recently been discovered, as several genome-wide association studies (GWAS) have identified SNPs in the *DHCR7* gene, as well as in the neighbouring *NADSYN1* gene, that are associated with 25(OH)D levels [54].

#### 3.3.3. Vitamin D Hydroxylases

All vitamin D-related CYP enzymes catalyse single or multiple hydroxylation reactions on specific vitamin D substrate carbons using a transient, heme-bound, oxidised iron intermediate compound (Fe-O) [24].

##### *CYP2R1* 

The *CYP2R1* gene encodes a key enzyme, microsomal vitamin D 25-hydroxylase, which converts vitamin D to 25(OH)D in the liver. The *CYP2R1* gene is located on the short arm of chromosome 11 at position 11p15.2. CYP2R1 catalyses the 25-hydroxylation of vitamin D2 and D3 at comparable rates and is the body’s primary source of 25(OH)D [55].

##### *CYP27A1* 

The primary role of CYP27A1 is the regulation of cholesterol and bile acid metabolism. The *CYP27A1* gene is located on chromosome 2 (2q35). Unlike *CYP2R1*, this gene hydroxylates only vitamin D3, whereas vitamin D2 undergoes hydroxylation at the 24th or 26th carbon atom, leading to the formation of alternative vitamin D metabolites [55,56].

##### *CYP27B1* 

The *CYP27B1* gene is located on the longer arm of chromosome 12 at position 14.1 (12q14.1). *CYP27B1* is present in kidney cells and produces the majority of circulating 1,25(OH)2D. *CYP27B1* is expressed in all types of bone tissue cells, in macrophages with associated inflammation, in activated dendritic cells and in glands whose secretion products are regulated by 1,25(OH)2D. Most CYP27B1 is synthesised in the kidney and regulated by almost every factor involved in calcium metabolism. Its activity is stimulated by PTH, calcitonin, calcium, GH and IGF-I, while it is inhibited by phosphorus, FGF-23 and, on a feedback basis, by 1,25(OH)D [57,58]. Current studies have shown that *CYP27B1* expression in macrophages is increased by cytokines rather than PTH [59].

##### *CYP24A1* 

The gene *CYP24A1* encodes 1,25(OH)2D-24-hydroxylase and is located on chromosome 20q13.2-q13.3. This enzyme acts on both calcidiol (25(OH)D) and calcitriol (1,25(OH)2D), leading to the formation of their inactive catabolites: 24,25(OH)2D and 1,24,25(OH)3D. CYP24A1 is expressed in tissues where the vitamin D receptor is present. The 24-hydroxylation reaction is an important mechanism to prevent vitamin D intoxication at the tissue level [60,61].

#### 3.3.4. *GC* (Group-Specific Component)

The majority of circulating 25(OH)D is bound to GC glycoprotein (vitamin D-binding protein, 83–85%), which is responsible for its transport from keratinocytes or enterocytes to the liver [47]. VDBP significantly prolongs the serum half-life of 25(OH)D3. Maintaining a constant amount of vitamin D metabolites modulates the degree of its bioavailability, activation and action on target organs. VDBP protects the body against short-term dietary vitamin D deficiencies [62]. VDBP is encoded by the *GC* gene located on chromosome 4q12-q13.

### 3.4. Nutritional Epigenomics

Each cell of an individual contains the same information necessary for the construction of proteins (i.e., the genome). However, in different cell types, the genome is packaged (condensed) by proteins into dense chromatin (heterochromatin) and less dense chromatin (euchromatin) in a specific way so that only those genes that carry information about proteins needed in particular tissues can be accessed [63]. Transcription factors and RNA polymerases can recognise genes in euchromatin. This genome packing does not affect the DNA sequence, i.e., it does not cause mutations [64]. The information regarding the genome contained in covalent modifications and chromatin structures constitutes the epigenome [65]. Chromatin plays an important role in the regulation of gene expression.

Epigenomics studies investigate chromatin changes that do not involve changes in the genome. Changes in the epigenome can affect the transcriptome (the total number of RNA molecules in cells). Many of the signals that affect the epigenome and transcriptome come from the diet. Hence, a significant aspect of nutrigenomics is to assess how nutrients affect the epigenome and transcriptome of cells and, thus, their function. This aspect of nutrigenomics is referred to as nutritional epigenomics [63].

Vitamin D and other dietary metabolites affect the activity of chromatin modifiers and transcription factors and, thus, chromatin accessibility. Hence, vitamin D is also included in nutritional epigenomics [64,66]. It has been shown that diet-induced epigenomic changes are often transient and reversible, in contrast to epigenomic programming [63].

Vitamin D influences the epigenome at multiple levels and plays an important role in the epigenetic regulation of genes. Epigenetic mechanisms may also regulate vitamin D metabolites [67]. Epigenetic modulation of vitamin D-related genes may cause vitamin D deficiency in the body.

### 3.5. Epigenetics of Vitamin D

#### 3.5.1. Effect of Vitamin D on DNA Methylation

The DNA methylation profile is altered by diet, SNPs in genes and exposure to environmental factors. DNA methylation is necessary to maintain cellular homeostasis and chromatin structure. Each of the genes encoding enzymes involved in the vitamin D metabolism pathway is regulated by DNA methylation. Methylation occurs at CpG sites (cytosine residues followed by guanine residues). The CpG clusters are CpG islands [8,68]. Differential methylation of these islands in gene promoter regions can affect gene expression, where hypermethylation is often associated with decreased expression and hypomethylation with increased expression [69]. The CYP2R1 methylation status regulates the effect of calcium and vitamin D intake or radiance on vitamin D serum levels: subjects presenting sufficient vitamin D levels, or taking vitamin D supplementation, show lower methylation at the CpG site of the *CYP2R1* gene [56]. Calcitriol can also affect DNA demethylation (most of which is passive demethylation), but the mechanisms of this action are unclear.

Hence, a complex and bidirectional relationship between DNA methylation and vitamin D metabolism is observed. Beckett et al. [68] showed that VDR methylation was positively correlated with plasma 25(OH)D levels and vitamin D intake. Increased VDR methylation in response to increased plasma 25(OH)D concentrations may reflect a potential negative feedback loop mechanism in which increased ligand availability reduces receptor expression to maintain vitamin D signalling homeostasis. Abnormal methylation and, thus, hypovitaminosis D, may contribute to an increased risk of certain diseases and impair the maintenance of normal calcium homeostasis. Furthermore, an inverse relationship was confirmed between CYP2R1 methylation levels and plasma 25(OH)D concentrations, while no such association was observed for CYP27B1. Since the relationship between plasma 25(OH)D and *CYP24A1* is only apparent in the absence of correction for vitamin D intake, this supports the hypothesis that altered *CYP24A1* methylation occurs in response to plasma 25(OH)D levels rather than that methylation levels are the factor explaining the deficiency [68].

#### 3.5.2. Effect of Vitamin D on Chromatin

Nuclear receptor–compressor complexes directly or indirectly catalyse the modification processes of histone proteins that are part of chromatin together with DNA (deacetylation, demethylation, dephosphorylation), leading to its condensation and repression of transcription. The VDR is among the best-characterised examples of transcriptional regulation in the context of chromatin [70]. Upon attachment of the heterodimer (VDR, RXR complex and VDRE sequence) to the DNA strand, protein complexes affecting chromatin structure with histone acetyltransferase (HAT) activity are released, including CREB-binding protein (CBP)/p300 and steroid receptor coactivator (SRC1) [71]. Transcription activation also relies on the action of chromatin remodelling complexes, which destabilise the junction between DNA strands and histones. After chromatin relaxation, VDR/RXR recruits the DRIP (VDR-interacting protein, mediator) multiprotein complex, which activates the transcription of target genes, directly increasing RNA polymerase II mobilisation and stabilising the transcription complex.

**Figure 5:**
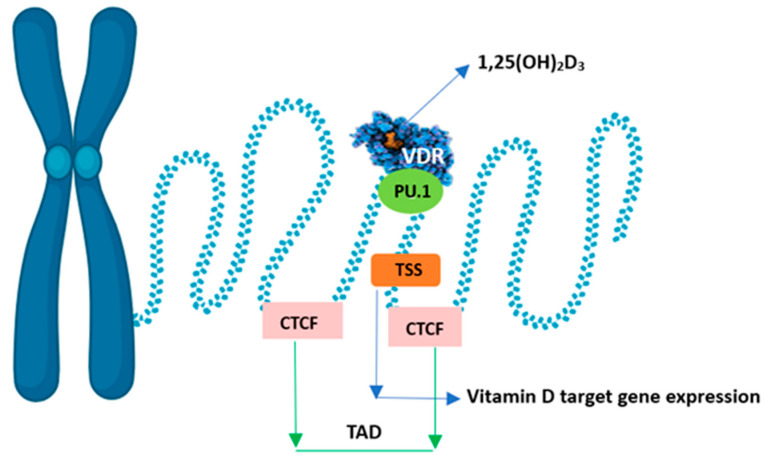
Chromatin model of vitamin D signalling.

The conformational change of the VDR-RXR dimer also releases corepressor proteins that mobilise histone deacetylases and DNA methyltransferases. This leads to strong chromatin condensation and blocks the promoter sequence of the gene in question, preventing access to transcription factors. These coregulators differ in their target tissue distribution and their specificity of action towards both 1,25(OH)2D and VDR. This leads to local changes in chromatin accessibility at multiple genomic sites, i.e., the epigenome responds to vitamin D. Chromatin accessibility is the main epigenetic determinant controlling gene expression [47]. The expression of the primary vitamin D target gene is modulated. An increase is observed chiefly when it co-occurs with a primary VDR-binding site within the same higher-order chromatin structure (topologically associated domain, TAD). In addition, the transcription start site (TSS) of the vitamin D target gene and the VDR-binding enhancer region must be within accessible chromatin. The genomic region that can be affected by VDR-binding calcitriol is limited by the CTCF proteins defining the left and right borders of the TAD. Hence, only vitamin D target genes within the TAD will be stimulated to produce more mRNA copies (Figure 5). CTCF is the main protein in the organisation of chromatin into active and inactive regions, but only 15% of CTCF sites in the whole genome are used to form TAD anchor regions. The pioneer factor, i.e., PU.1, is a transcription factor that acts as the first enhancer by binding proteins that interact with chromatin-remodelling enzymes and is thus more accessible to, for example, the VDR [72].

The more distant the VDR-binding sites are from the TSS, the less likely they are to be functional for the gene. In addition, the VDR-binding site in the enhancer region and the TSS of the vitamin D receptor-controlled target gene must be in the same TAD [34]. Changes in the epigenome are the first events after stimulation of the cell with vitamin D before the transcript is modulated. Equal distribution of permanent VDR-binding sites in the human genome has been shown to contribute to whole-genome vitamin D sensitivity [73].

#### 3.5.3. Histone Modifications

Nucleosomes are constructed from eight histone proteins rich in lysine and arginine. These amino acids are often post-translationally modified by methyl and acetyl groups. Hence, one of the main components of epigenetic changes is the reversible post-translational modification of histone proteins (acetylation and methylation) directed by chromatin-modifying enzymes, viz., HAT, HDAC, HMT and HDM. Post-translational modifications of histones correlate with active or inactive regions of chromatin, altering its structure [74].

#### 3.5.4. MicroRNA

Dietary components can affect the expression of microRNAs (miRNAs) and modify their function. Evidence suggests that miRNAs in food can also be absorbed during the digestive process and consequently affect host gene expression [75]. The possibility of using miRNAs as biomarkers for various diseases is being tested. In addition, miRNAs are important mediators of vitamin D signalling. miRNAs are a class of small non-coding RNAs (ncRNAs) of ~18 to 22 nucleotides that play an important role in post-transcriptional regulation of gene expression and gene silencing [76]. A single miRNA can interact with several genes, making it a very efficient mechanism for influencing biological responses. More than 1700 human miRNAs have been identified, affecting about 60% of genes, but most of these miRNAs’ functions and target genes are still unknown [77].

miRNAs regulate gene expression by directly binding to a complementary sequence in the 3′-encoding region (3′-UTR) of mRNA, causing mRNA degradation or repression of translation [76]. Not only do miRNAs affect vitamin D signalling, but vitamin D also regulates miRNA networks during homeostasis and disease [78]. Vitamin D can affect miRNA gene transcription by binding the VDR to its sequence motif located in the promoter of miRNA target genes.

Vitamin D increases levels of specific miRNAs and regulates miRNA expression globally through VDR-dependent chromatin opening and increased pri-miRNA expression [79]. Several miRNAs have been shown to regulate several genes involved in the vitamin D pathway, such as VDR, CYP24A1, CYP27B1 and RXRα. Four miRNAs, namely miR-125b, miR-27b, miR-298 and miR-346, were detected targeting the VDR. Lisse et al. [80] showed that miRNAs play a key role in tuning the effects of 1,25(OH)2D on osteoblast differentiation and function.

Elucidating the role of miRNAs in osteoblast regulation may provide new strategies to study the pathogenesis and treatment of vitamin D-related bone diseases such as rickets and osteoporosis. Studies using different cancer cells have demonstrated the induction of miRNAs by vitamin D. The first miRNA targeting the VDR was identified by Mohri et al. [78], who showed that miR-125b directly regulates the expression of the *VDR* gene in the breast cancer cell line MCF-7 and that its overexpression can abrogate the antiproliferative effect of vitamin D. Furthermore, miR-27b is a regulator of *VDR* gene expression in melanoma, colorectal cancer and pancreatic cancer cell lines [81,82]. *CYP24A1* is regulated by miR-125b [83].

It has been investigated that an active vitamin D metabolite suppresses the expression of human telomerase reverse transcriptase (hTERT) and the growth of the human ovarian cancer cell line OVCAR3 through the induction of miR-498 in a vitamin D-dependent manner [84] and miR-98 is a key mediator of the antiproliferative effect of vitamin D in prostate cancer [85].

Beckett et al. [86] demonstrated an association between serum circulating let-7a/8 microRNA levels and vitamin D intake, which was dependent on the *VDR* gene allele for the BsmI (rs1544410) and ApaI (rs7975232) single-nucleotide polymorphisms. The analysis demonstrated the importance of considering genotypic variants in the vitamin D-related *VDR* gene in studies focusing on miRNA expression and serum vitamin D levels. In summary, a correlation was found between epigenetic modulations of vitamin D and genome variation and the importance of assessing human genome variation, which may be responsible for differences in responses to vitamin D treatment [86]. Hence, inter-individual genome variability in miRNA—and vitamin D-related genes should be taken into account when interpreting results, as the presence of single-nucleotide polymorphisms may result in different responses to vitamin D.

### 3.6. Polymorphisms of Vitamin D-Related Genes

Many candidate genes and genome-wide association studies have already been carried out. From these, several genetic mutations and polymorphisms affecting genes encoding molecules involved in vitamin D production and activation, transport proteins, VDR and other proteins involved in the regulation of vitamin D expression have been demonstrated.

The most studied candidate genes include *VDR, DHCR7, CYP2R1, CYP27B1, GC* and *CYP24A1* [87,88].

It has been speculated that SNPs in these genes may affect their function or expression and thus affect the biological activity of vitamin D. Consequently, these changes can be considered as predictive biomarkers of disease [89].

#### 3.6.1. *VDR* Gene Polymorphism

Polymorphisms occur within the VDR receptor gene. Due to the involvement of vitamin D in cell differentiation and death, as well as in limiting the number of cell divisions, the polymorphisms, viz., TaqI (rs731236, C > T), BsmI (rs1544410, A > G), FokI (rs2228570, T > C), ApaI (rs7975232, A > T) and Cdx2 (rs11568820, A > G) may be important in the origin of many diseases: cancer, metabolic diseases including obesity, cardiovascular, neurodegenerative and autoimmune diseases. Furthermore, their presence correlates with reduced bone density and increased susceptibility to infection [90,91].

The FokI polymorphism is located in the coding part at the START codon—the polymorphic form results in a protein that is three amino acids shorter. The BsmI, TaqI and ApaI polymorphisms are located in the part of the 3’UTR sequence responsible for the stability of the resulting mRNA. BsmI and ApaI are located in intron 8 of the gene, and TaqI in exon 9 [92]. Only FokI has a negligible effect on vitamin D levels. Although genetic variants in the *VDR* do not appear to have a significant effect on 25(OH)D levels, they significantly affect *VDR* expression and its independent function. A correlation between the methylation status of the *VDR* gene and serum vitamin D levels has been shown [67].

*Cdx2* genotypes differ significantly in their primary vitamin D metabolite levels, 25(OH)D3. For the *Cdx2* polymorphism, a predisposition to osteoporosis and cancer development has been found. There are also reports on the effect of the Cdx2 SNP of the *VDR* gene on immune function [93].

#### 3.6.2. *NADSYN1/DHCR7* Gene Polymorphism

Since there is insufficient UVB radiation to induce year-round cutaneous synthesis of vitamin D at latitudes distant from the equator, it is likely that these genes were subject to forces of natural selection. Genetic variation in *DHCR7* is the major adaptation affecting vitamin D metabolism in recent evolutionary history which helped early humans to avoid severe vitamin D deficiency and enabled them to inhabit areas further from the equator [54]. This implies that *DHCR7* mutations associated with higher vitamin D status conferred a survival advantage which allowed early humans to avoid severe deficiency when migrating to northern latitudes. This suggests that mutations associated with higher vitamin D status were positively selected at higher latitudes in both continents over different periods due to a variety of environmental pressures, as they proffered an evolutionary advantage. These genetic variants then rose quickly to high frequencies in humans living in areas distant from the equator. The differences in timing of the selective events between the European and Northeast Asian populations may in part reflect the diets of the respective populations in the late Pleistocene and early Holocene. These studies show that humans and animals have adapted mechanisms to exploit optimal methods of synthesising vitamin D in response to their environment. The increased frequencies of *DHCR7* alleles associated with higher vitamin D status in the hypopigmented populations of Europe and Northeast Asia may represent yet another adaptation which conferred a survival advantage allowing early humans to avoid severe deficiency when migrating to northern latitudes [54].

GWAS studies in populations of European origin identified associations for several novel variants (rs3829251, rs12785878 and rs1790349) near *NADSYN1* and *DHCR7*. Risk alleles of common variants at the *NADSYN1/DHCR7* loci were significantly associated with reduced plasma 25(OH)D levels. Individuals with the rs3829251 AA and rs12785878 GG genotypes were shown to have lower 25(OH)D levels on average compared to other genotypes [94,95].

#### 3.6.3. *CYP2R1* Gene Polymorphism

Genetic variation in the *CYP2R1* gene can affect 25(OH)D synthesis. Among the most commonly analysed polymorphisms is rs10741657, where there is an A > G change in the 5’ UTR region responsible for translation initiation and mRNA stabilisation. A meta-analysis conducted by Duan et al. [96] confirmed a statistically significant association between SNP rs10741657 and 25(OH)D levels and vitamin D deficiency in Caucasian and Asian populations. Ramos-Lopez et al. [97] also showed an association between SNP rs10741657 and 25(OH)D levels in people with type 1 diabetes, where those carrying the AA genotype had higher 25(OH)D levels compared with those with the GG and GA genotypes, with a difference of up to 14 ng/mL between the genotypes. The presence of the G allele and the GG and GA genotypes correlates with an increased risk of vitamin D deficiency [97]. It was shown that individuals with the AA genotype were 2.5 times more likely to have increased serum 25(OH)D levels after high-dose vitamin D supplementation (50,000 IU/week) than those with the GG genotype. Thus, the rs10741657 variant of the *CYP2R1* gene modulates the response to high-dose vitamin D supplementation [98].

Nissen et al. [99] showed that *CYP2R1* gene variants, i.e., rs1562902, rs7116978 and rs10766197, were significantly associated with serum 25(OH)D concentrations in children and adults. Non-carriers of the risk alleles rs10741657 and rs10766197 had the highest mean serum 25(OH)D concentrations. Multiple SNP variants in the *CYP2R1* gene have also been linked to several chronic diseases, including obesity, asthma, type 2 diabetes and cancer [100]. CYP2R1 enzyme expression is modulated by age and metabolic environment. 25(OH)D levels decrease and are less sensitive to supplementation in older people. This is most likely related to the decreasing activity of CYP2R1 during the ageing process [20].

#### 3.6.4. *CYP27B1* Gene Polymorphism

CYP27B1 is the cytochrome P450 most strongly associated with vitamin D status. Due to its key role in vitamin D metabolism, polymorphisms and mutations within *CYP27B1* can lead to significant changes in the levels of this vitamin in the body and underlie certain conditions. In the case of the rs10877012 polymorphism (C > A), the presence of the C allele has been shown to be associated with lower serum 25(OH)D levels in Caucasian and African–American individuals [101]. According to Hu et al. [102], the rs10877012 polymorphism does not affect baseline vitamin D levels in people with type 2 diabetes, but GT heterozygotes respond better to vitamin D supplementation compared with GG and TT homozygotes.

#### 3.6.5. *CYP24A1* Gene Polymorphism

Vitamin D metabolism is also significantly dependent on the *CYP24A1* gene encoding 24-hydroxylase. This enzyme deactivates the active form of vitamin D to metabolites with little biological activity, such as 24,25(OH)2D through oxidation reactions, thus preventing its toxicity [103]. Polymorphisms in *CYP24A1*, i.e., rs2585428 and rs4809960, have been shown to lead to changes in vitamin D catabolism and homeostasis. A 10-fold increase in the half-life of calcitriol, resulting in hypervitaminosis, hypercalcaemia, hypercalciuria and lower PTH levels, was observed [104]. These changes indicate a key role for *CYP24A1* in maintaining vitamin D balance in the body.

#### 3.6.6. *GC* Gene Polymorphism

The *GC* gene encodes a VDBP protein that binds and transports 25(OH)D in the blood and other vitamin D metabolites to their target organs. Although the *GC* gene is highly polymorphic, two primary polymorphisms, rs4588 (C > A) and rs7041 (G > T), have been shown in most humans to produce three major *GC* isoforms: GC1F (rs7041-T, rs4588-C), GC1S (rs7041-G, rs4588-C) and GC2 (rs7041-T, rs4588-A) dependent on race and skin pigmentation [105]. GC2 is most commonly present in Caucasians, while the GC1F type of vitamin D-binding protein is found in Black people [106].

*GC* gene variations may affect VDBP binding and 25(OH)D bioavailability. Thus, there may be a relationship between phenotype and blood 25(OH)D concentrations [99,107].

It was shown that individuals with the rs4588C allele had lower serum vitamin D levels [108]. Individuals with the rs7041G allele have also been shown to have an increased risk of vitamin D deficiency. In men, this risk was higher than in women [103].

### 3.7. Personalisation of Vitamin D Supplementation in Response to Genetic Differences–Future Directions

The level of 25(OH)D as an indicator of vitamin D status in the body is determined by the availability of dermal synthesis under sunlight and the contribution of diet and supplement intake. Vitamin D status is also determined by genetic variation. GWAS studies have shown many variants associated with serum 25(OH)D concentrations and may influence the prevalence of vitamin D deficiency [109].

Hence, serum 25(OH)D levels change after vitamin D supplementation and may vary significantly between individuals. Determinants such as age, sex, body weight, type of supplementation, calcium intake, baseline 25(OH)D concentration and physical activity contribute to 50% of the modulation of calcidiol levels [110]. Genetic variability and epigenetics are responsible for the remainder. It has previously been suggested that individuals with high methylation rates of *CYP2R1* and *CYP24A1* genes may need higher doses of vitamin D supplementation to achieve optimal serum levels [111].

A new concept called personalised response to vitamin D supplementation has been put forward [112]. The large-scale studies VitDbol (NCT02063334) and VitDmet (NCT01479933) showed significant differences in the molecular response of people to vitamin D supplementation [111,112]. Due to the individual response to the vitamin, people were divided into strong, moderate and weak responders [63]. A low response rate to vitamin D was found in 25% of the study participants. Such individuals are most at risk of vitamin D deficiency. Therefore, they should take significantly higher daily doses of vitamin D compared to high responders to achieve optimal vitamin D hormonal activity and maximum protection against vitamin D deficiency-related diseases. Interestingly, the vitamin D response rate is independent of serum 25(OH)D levels. Hence, there are individuals with a high response to low vitamin D status and individuals with a low response to supplementation with high vitamin D status. High responders can cope with low 25(OH)D levels, whereas low responders need high vitamin D status to benefit from it [63,113].

## 4. Conclusions

The field of nutrigenomics faces a significant challenge in the coming years: to understand better how diet and its bioactive components influence gene expression and key metabolic and signalling pathways. Vitamin D provides a compelling example of such interaction. Interindividual variability in response to vitamin D supplementation can be partly explained by person-specific sets of vitamin D target genes and their epigenetic sensitivity.

Vitamin D interacts with the epigenome at multiple levels, including DNA methylation, histone modifications and microRNA regulation. These mechanisms may account for variations in vitamin D metabolism and circulating levels across populations. However, the full impact of these epigenetic modifications on vitamin D bioavailability, metabolic outcomes and long-term health remains to be elucidated.

A deeper understanding of the molecular pathways of vitamin D action—through both in vitro and in vivo models—is essential for optimising its clinical use. Low vitamin D status may be a biomarker of increased susceptibility to metabolic, immune or inflammatory disorders, highlighting its relevance for preventive medicine and public health.

Integrating epigenetic profiling into clinical practice may pave the way for more precise, safe and effective vitamin D supplementation strategies. Ultimately, exploring the epigenetic regulation of vitamin D contributes to the broader goal of developing truly personalised nutritional interventions.

In this context, vitamin D emerges as a model nutrient at the intersection of nutrigenomics and epigenetics, illustrating how molecular nutrition can shape the future of personalised health.

## Data Availability

No new data were created or analysed in this study. Data sharing is not applicable to this article.
